# Peak width of skeletonized mean diffusivity in cerebral amyloid angiopathy: Spatial signature, cognitive, and neuroimaging associations

**DOI:** 10.3389/fnins.2022.1051038

**Published:** 2022-11-11

**Authors:** Maria Clara Zanon Zotin, Dorothee Schoemaker, Nicolas Raposo, Valentina Perosa, Martin Bretzner, Lukas Sveikata, Qi Li, Susanne J. van Veluw, Mitchell J. Horn, Mark R. Etherton, Andreas Charidimou, M. Edip Gurol, Steven M. Greenberg, Marco Duering, Antonio Carlos dos Santos, Octavio M. Pontes-Neto, Anand Viswanathan

**Affiliations:** ^1^J. Philip Kistler Stroke Research Center, Department of Neurology, Massachusetts General Hospital, Harvard Medical School, Boston, MA, United States; ^2^Center for Imaging Sciences and Medical Physics, Department of Medical Imaging, Hematology and Clinical Oncology, Ribeirão Preto Medical School, University of São Paulo, Ribeirão Preto, Brazil; ^3^Department of Psychiatry, Harvard Medical School, Massachusetts General Hospital, Boston, MA, United States; ^4^Toulouse NeuroImaging Center, Université de Toulouse, Inserm, UPS, Toulouse, France; ^5^German Center for Neurodegenerative Disease, Magdeburg, Germany; ^6^University of Lille, Inserm, CHU Lille, U1172 - LilNCog (JPARC) - Lille Neurosciences & Cognition, Lille, France; ^7^Division of Neurology, Department of Clinical Neurosciences, Geneva University Hospitals, Geneva, Switzerland; ^8^Institute of Cardiology, Medical Academy, Lithuanian University of Health Sciences, Kaunas, Lithuania; ^9^The First Affiliated Hospital of Chongqing Medical University, Chongqing, China; ^10^Department of Neurology, Boston University School of Medicine, Boston University Medical Center, Boston, MA, United States; ^11^Department of Biomedical Engineering, Medical Imaging Analysis Center (MIAC), University of Basel, Basel, Switzerland; ^12^Department of Neuroscience and Behavioral Sciences, Ribeirão Preto Medical School, University of São Paulo, Ribeirão Preto, Brazil

**Keywords:** cerebral amyloid angiopathy, cerebral small vessel disease, dementia, vascular cognitive impairment, diffusion tensor imaging, diffusion-weighted imaging

## Abstract

**Background:**

Peak width of skeletonized mean diffusivity (PSMD) is a promising diffusion tensor imaging (DTI) marker that shows consistent and strong cognitive associations in the context of different cerebral small vessel diseases (cSVD).

**Purpose:**

Investigate whether PSMD (1) is higher in patients with Cerebral Amyloid Angiopathy (CAA) than those with arteriolosclerosis; (2) can capture the anteroposterior distribution of CAA-related abnormalities; (3) shows similar neuroimaging and cognitive associations in comparison to other classical DTI markers, such as average mean diffusivity (MD) and fractional anisotropy (FA).

**Materials and methods:**

We analyzed cross-sectional neuroimaging and neuropsychological data from 90 non-demented memory-clinic subjects from a single center. Based on MRI findings, we classified them into probable-CAA (those that fulfilled the modified Boston criteria), subjects with MRI markers of cSVD not attributable to CAA (presumed arteriolosclerosis; cSVD), and subjects without evidence of cSVD on MRI (non-cSVD). We compared total and lobe-specific (frontal and occipital) DTI metrics values across the groups. We used linear regression models to investigate how PSMD, MD, and FA correlate with conventional neuroimaging markers of cSVD and cognitive scores in CAA.

**Results:**

PSMD was comparable in probable-CAA (median 4.06 × 10^–4^ mm^2^/s) and cSVD (4.07 × 10^–4^ mm^2^/s) patients, but higher than in non-cSVD (3.30 × 10^–4^ mm^2^/s; *p* < 0.001) subjects. Occipital-frontal PSMD gradients were higher in probable-CAA patients, and we observed a significant interaction between diagnosis and region on PSMD values [*F*(2, 87) = 3.887, *p* = 0.024]. PSMD was mainly associated with white matter hyperintensity volume, whereas MD and FA were also associated with other markers, especially with the burden of perivascular spaces. PSMD correlated with worse executive function (β = −0.581, *p* < 0.001) and processing speed (β = −0.463, *p* = 0.003), explaining more variance than other MRI markers. MD and FA were not associated with performance in any cognitive domain.

**Conclusion:**

PSMD is a promising biomarker of cognitive impairment in CAA that outperforms other conventional and DTI-based neuroimaging markers. Although global PSMD is similarly increased in different forms of cSVD, PSMD’s spatial variations could potentially provide insights into the predominant type of underlying microvascular pathology.

## Introduction

Cerebral amyloid angiopathy (CAA) is a form of cerebral small vessel disease (cSVD) related to the deposition of amyloid-beta around cortical and leptomeningeal vessels (Viswanathan and Greenberg, 2011). CAA is highly prevalent among older individuals and impacts cognition independently from commonly co-occurring Alzheimer’s disease (AD) (Arvanitakis et al., 2011). CAA has thus emerged as an important vascular contributor to cognitive impairment and dementia (VCID), even in the absence of intracerebral hemorrhage (ICH) (Xiong et al., 2017).

In order to facilitate the future development of disease-modifying therapies for VCID, it has become paramount to validate neuroimaging markers able to quantify the widespread parenchymal injury associated with cSVD (Smith et al., 2019). Among the many MRI markers available, those based on diffusion tensor imaging (DTI) provide more consistent cognitive associations due to their continuous quantitative nature and higher sensitivity to microstructural abnormalities (Smith and Beaudin, 2018).

A novel DTI marker called peak width of skeletonized mean diffusivity (PSMD) is considered particularly promising in the field of VCID (Baykara et al., 2016). PSMD is a fully automated histogram marker that reflects the heterogeneity of the mean diffusivity (MD) values across the main white matter (WM) tracts. It was developed to quantify the WM injury related to VCID and has provided consistent cognitive associations in several populations, especially among cohorts with cSVD (Baykara et al., 2016; Deary et al., 2019; Lam et al., 2019; Wei et al., 2019; Low et al., 2020; McCreary et al., 2020; Raposo et al., 2021). According to previous data, PSMD correlates with processing speed scores in CAA samples and could become a relevant marker for CAA-related cognitive impairment (McCreary et al., 2020; Raposo et al., 2021).

However, several questions concerning the utility of this marker in the field of CAA remain unanswered. In general, PSMD values found in samples with cSVD are higher than those with predominantly neurodegenerative pathology. However, it remains unknown whether PSMD values vary significantly across different forms of sporadic cSVD. Regional analyses with PSMD are thought to be feasible (Beaudet et al., 2020), and lobe-specific PSMD values have been successfully computed in a previous study (Low et al., 2020). However, no studies to date have investigated spatial variations in PSMD values in the context of CAA. Specifically, it is unknown whether PSMD could capture the posterior predominance of CAA pathology, like other diffusion techniques (Reijmer et al., 2015a). Furthermore, only one study in CAA has compared PSMD with other classical DTI markers in terms of cognitive and neuroimaging associations (McCreary et al., 2020), but the extent to which this new marker outperforms MD and fractional anisotropy (FA) remains uncertain.

In this context, our aims were: (1) to compare PSMD values in different MRI phenotypes of sporadic cSVDs and in subjects without evidence of cSVD on MRI; (2) to investigate whether the posterior predominance of CAA pathology is reflected on regional variations of PSMD values; (3) to investigate PSMD’s cognitive and neuroimaging associations in CAA, compared to other DTI-based MRI markers.

## Materials and methods

### Study participants

This study is a retrospective analysis of an ongoing single-center prospective memory-clinic research cohort from the MGH. Subjects were recruited between August 2010 and November 2019.

We included non-demented subjects aged 55 years or more with complete clinical evaluation, neuropsychological examination, and 3 T MRI. Exclusion criteria were: dementia (defined as Mini Mental State Examination [MMSE] ≤ 24 and/or impairment of independent activities of daily living [IADs]); a history of symptomatic intracerebral hemorrhage (ICH); incomplete neuropsychological examination and/or research MRI; and presence of motion or other artifacts compromising neuroimaging assessment. To avoid diagnostic uncertainty, we also excluded subjects with a single hemorrhagic MRI marker fulfilling the modified Boston criteria (Linn et al., 2010) for possible-CAA and those with mixed-pattern of hemorrhages (Pasi et al., 2018). Based on the examination of MR images for conventional neuroimaging markers of cSVD, and according to the modified Boston criteria (Linn et al., 2010), we stratified the participants into three groups: probable-CAA (patients fulfilling Boston criteria for probable CAA); cSVD subjects not fulfilling criteria for either possible or probable CAA and presenting one or more of the other following MRI markers of cSVD: non-lobar cerebral microbleeds [CMB], and/or lacunes, and/or high-grade (>2) basal ganglia perivascular spaces (PVS) (Potter et al., 2015), and/or deep Fazekas score (Fazekas et al., 1987) ≥ 2 and/or periventricular Fazekas score [Fazekas et al. (1987) = 3]; and non-cSVD (subjects without any of the above neuroimaging markers of cSVD). None of the cSVD subjects had any MRI or clinical features suggesting a hereditary/monogenic pathology. Therefore, they are presumably affected by the most prevalent etiological subtype of sporadic cSVD, related to aging and/or vascular risk factors such as hypertension (arteriolosclerosis/deep perforator arteriopathy).

### Clinical and neuropsychological assessment

Demographic and clinical data were collected from each participant upon enrollment. All memory-clinic subjects underwent a thorough and standardized neuropsychological test battery. We explored four different cognitive domains through composite scores created by clustering and averaging performance on different neuropsychological tests (Weintraub et al., 2009): Executive function [Trail Making Test B (Corrigan and Hinkeldey, 1987) and Digit Span Backward (Joy et al., 2004)] processing speed/attention [Trail Making Test A (Sánchez-Cubillo et al., 2009), Digit Span Forward (Joy et al., 2004), and WAIS-III (Wechsler Adult Intelligence Scale-Third Edition) Digit Symbol Coding (Joy et al., 2004)], memory [Hopkins Verbal Learning Test-Revised (Brandt, 1991) and Wechsler Memory Scale logical memory (Wechsler, 1987); immediate recall and delayed recall], and language function [Boston Naming Test (Mack et al., 1992) and Animal Naming (Tombaugh et al., 1999)]. First, based on published normative data (Crum et al., 1993; Fastenau et al., 1998; Tombaugh, 2004), we transformed performance on each test into z-scores adjusted for gender, age, and education level. Then, these z-scores were averaged within composite domains to compute the domain-specific scores for each participant.

### MRI acquisition

All exams were performed on a 3-T MRI scanner (Siemens Healthcare, Magnetom Prisma-Fit, or TIM-Trio), using a 32-channel head coil. The scan protocol included: a 3D T1-weighted sequence (repetition time [TR] 2,300–2,510 ms; echo time [TE] 1.69–2.98 ms; resolution 1 × 1 × 1 mm), a 3D fluid-attenuated inversion recovery sequence (FLAIR, TR 5,000–6,000 ms; TE 356–455 ms; resolution 1 × 1 × 1 mm), a susceptibility-weighted imaging sequence (SWI, TR 27–30; TE 20; slice thickness, 1.4 mm; in-plane resolution 0.9 × 0.9 mm), and a 3D multi-directional diffusion-weighted imaging sequence (DWI, 60–64 directions; TR 8,000–8,040 ms; TE 82–84 ms; resolution 2 × 2 × 2 mm; b-value, 700 s/mm^2^).

### Peak width of skeletonized mean diffusivity processing

Initially, a careful visual inspection of all DWI images was conducted, and cases with excessive motion artifacts were excluded. To objectively and quantitatively evaluate the magnitude of head motion during the acquisition of DWI images, we further extracted registration- and intensity-based measures to compute the Total Motion Index (TMI) proposed by Yendiki et al. (2014). TMI was originally developed to be used as a nuisance regressor to account for motion confounding in neuroimaging studies (Yendiki et al., 2014). Since the intensity-based measures did not show significant variability across our subjects, only the registration-based measures were applied in the TMI formula.

We ran the fully automated PSMD script (version 1.0/2016)^1^ (Baykara et al., 2016), including all the pre-processing steps, relying on the Functional Magnetic Resonance Imaging of the Brain (FMRIB) Software Library (FSL) version 6.0.1 (Jenkinson et al., 2012). Briefly, the script runs motion and eddy-currents correction (Andersson and Sotiropoulos, 2015), brain extraction (Smith, 2002), and tensor fitting as pre-processing steps, obtaining maps of MD and FA. These maps are fed into tract-based spatial statistics (TBSS), as implemented in FSL (Smith et al., 2006), to achieve skeletonization of the main WM tracts. The obtained skeletonized MD maps are further masked with a custom mask to exclude areas prone to CSF contamination. Finally, a histogram analysis is conducted on the final MD maps, and the difference between percentiles 5 and 95 is computed to obtain PSMD values for each individual. Similar procedures were used to compute skeletonized average MD (McCreary et al., 2020) and FA values.

### Regional distribution of peak width of skeletonized mean diffusivity

**FIGURE 1:**
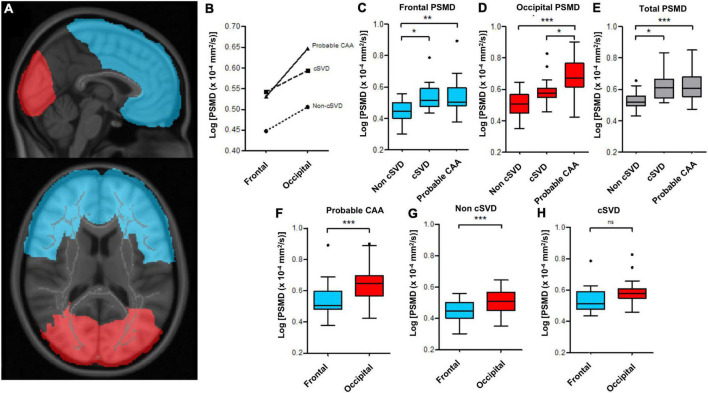
Regional differences in PSMD values between probable-CAA, cSVD and non-cSVD groups. **(A)** Frontal (blue) and occipital (red) masks, used to compute lobe-specific PSMD values. **(B)** The line graph depicts mean log-PSMD values in the frontal and occipital lobes of probable-CAA (solid line), cSVD (dashed line), and non-cSVD (dotted line) subjects. **(C–H)** Box-plots of PSMD values. The boxes extend from the 25th to 75th percentiles, and the solid line within each box represents the median. The superior and inferior inner fences represent the 75th percentile plus 1.5 times the interquartile range and the 25th minus 1.5 times the interquartile range, respectively. Outliers are displayed by symbols. **(C**–**E)** Box plots contrasting frontal **(C)**, occipital **(D)**, and total **(E)** PSMD values between non-cSVD, cSVD, and probable-CAA groups. **(F–H)** Box-plots display frontal (blue) and occipital (red) PSMD within each study group. General linear models adjusted for age are indicated by the brackets, with *p*-values (*p < 0.05; ** *p* < 0.01; *** *p* < 0.001).

To investigate potential differences in the white matter microstructural integrity across the anteroposterior axis, we used MNI152 atlas to create masks of the frontal and occipital lobes, manually filling missing voxels in the deep and periventricular WM areas (Figure 1A).

We applied these two masks separately to all subjects and computed PSMD, skeletonized average MD, and skeletonized average FA values restricted to the frontal and occipital lobes. To depict variations across the anteroposterior axis, we computed occipital-frontal gradients (occipital—frontal DTI metrics) for each participant.

### Conventional neuroimaging markers

Conventional MRI markers of cSVD were rated by a neuroradiologist (M. C. Z. Z.), blinded to all clinical data, following the Standards for Reporting Vascular Changes on Neuroimaging (STRIVE) recommendations (Wardlaw et al., 2013). CMBs were evaluated on SWI for presence, number, and location (Wardlaw et al., 2013). Lacunes were evaluated on FLAIR images as lesions measuring 3–15 mm, isointense to CSF, with hyperintense margins (Wardlaw et al., 2013). We evaluated SWI for the presence of cortical superficial siderosis (cSS) (Linn et al., 2010). Cortical cerebral microinfarcts (CMIs), defined as lesions ≤4 mm, restricted to the cortex, hypointense on T1, and hyperintense on FLAIR, were counted across the whole brain (van Veluw et al., 2017). We rated PVS in the centrum semiovale (CSO-PVS) and the basal ganglia (BG-PVS), using T1-weighted images, according to a previously proposed scale: 0 (none); 1 (1–10); 2 (11–20); 3 (21–40); 4 (>40) (Potter et al., 2015). White matter hyperintensities (WMH) were qualitatively assessed using a validated visual scale (Fazekas et al., 1987).

We used the *FreeSurfer* software suite (version 6.0)^2^ (Dale et al., 1999) to compute total brain volume (TBV) and total intracranial volume (ICV), following a rigorous visual quality inspection. The normalized TBV (nTBV) was calculated as the TBV/ICV ratio multiplied by 100, and was expressed in units of percent ICV. We used the lesion prediction algorithm (Schmidt, 2017) implemented in the toolbox Lesion Segmentation Tool version 3.0.0^3^ for Statistical Parametric Mapping 12 to segment WM hyperintense lesions, following visual inspection for segmentation quality. Different lesion probability thresholds were evaluated on 40 randomly selected individuals from our cohort, and the ideal threshold of 0.5 was defined by careful visual inspection against their respective FLAIR images for accuracy. We calculated normalized WMH volume (nWMHV) as the WMHV/ICV ratio multiplied by 100 and expressed in units of percent ICV.

### Statistical analysis

The distribution of continuous variables was tested for normality using the Shapiro-Wilk test. Clinical, neuropsychological, and neuroimaging characteristics were compared across the three groups using χ2 or Fisher test and Kruskal-Wallis H test or ANOVA, as appropriate.

We investigated the association between TMI and DTI metrics across all subjects, using simple linear regression, further adjusting for age, gender, and education level to evaluate motion effects. Neuroimaging and cognitive associations were investigated only in the probable CAA group. These analyses were restricted to the probable CAA group because we intended to investigate PSMD’s performance specifically in the context of the second most common form of sporadic cSVD, for which there is still limited data available in the literature. Linear regression models exploring the associations between DTI metrics and conventional neuroimaging markers were adjusted for age, and those investigating cognitive associations were adjusted for the time interval between the MRI and the neuropsychological tests. Since cognitive scores had already been adjusted for age, gender, and education level, we did not include these variables in the models. We further assessed the relative contribution of each neuroimaging variable by running multiple linear regression models and applying a decomposition method proposed by Lindeman et al. (1980) and available in the Relaimpo R package (v2.2) (Grömping, 2006).

Non-parametric variables were log-transformed for the regional analyses. To investigate regional variances in PSMD, MD, and FA we compared occipital-frontal gradients between the groups, using ANCOVA, adjusting for age. Furthermore, to evaluate whether CAA diagnosis could influence regional variances in DTI metrics, we compared probable-CAA subjects against non-cSVD and cSVD subjects. We used mixed-factor ANOVA to investigate whether there was an interaction between group and region on PSMD, MD, and FA values. We further investigated the simple main effects of group and region using ANCOVA adjusted for age and repeated-measures ANOVA, respectively. All pairwise comparisons were adjusted for false discovery rate (FDR) using the Benjamini-Hochberg procedure.

The statistical significance level was set at 0.05 for all analyses. We used the Statistical Package for the Social Sciences (SPSS) version 20.0 (for IOS; SPSS Inc., Chicago, IL), Prism (v8.4.3), and R (v3.5.3) to run the analyses.

## Results

We screened 167 subjects from our memory-clinic cohort who underwent complete research MRI and neuropsychological exam. Of those subjects, 77 were excluded, based on the following pre-specified criteria: dementia (*n* = 46), excessive motion artifacts on visual inspection (*n* = 3); possible-CAA (*n* = 26); and mixed pattern of distribution of hemorrhagic features (*n* = 2). The final cohort was comprised of 90 non-demented memory-clinic subjects (43 probable-CAA, 17 non-CAA-cSVD and 30 non-cSVD). The median delay between MRI and neuropsychological tests was 0 [IQR, 0–2.5] months.

In simple linear regression analysis, TMI was not association with any DTI metric (Standardized β coefficient [95% confidence interval]; PSMD β [95% CI] = −0.027 [−0.239, 0.185], *p* = 0.802; MD β [95% CI] = −0.038 [−0.250, 0.173], *p* = 0.721; FA β [95% CI] = 0.051 [−0.160, 0.263], *p* = 0.632), even after adjusting for age, gender and education level. Therefore, we did not include TMI as a covariate in the neuroimaging and cognitive analyses.

### Between-group comparisons

Demographic, neuropsychological, and neuroimaging data from each group are summarized in Table 1. Groups differed in age [*F*(2, 87) = 9.326, *p* < 0.001], with both probable-CAA (mean difference ± standard error, 4.67 ± 1.52; FDR-adjusted *p*-value = 0.004) and cSVD (7.95 ± 1.94; *p* < 0.001) being older than non-cSVD subjects. Gender, education level, vascular risk factors, cognitive performance, and head motion (TMI) were evenly distributed across the groups. As expected, the burden of cSVD markers was higher in the probable-CAA and cSVD groups compared to the non-cSVD group. nTBV differed across the groups [*F*(2, 87) = 8.905, *p* < 0.001], with greater atrophy in probable CAA subjects than in non-cSVD participants (−3.94 ± 0.93; *p* < 0.001). PSMD values also differed across the groups, and were lower in the non-cSVD group in comparison to probable-CAA (*p* < 0.001) and cSVD (*p* < 0.001). PSMD did not differ between probable-CAA and cSVD groups (*p* = 0.883). On the other hand, MD values were higher in probable-CAA patients, and FA values were lower in CAA patients, compared to both cSVD and non-cSVD groups (*p* < 0.001).

**TABLE 1:** Summary of clinical and neuroimaging data.

	Total (*n* = 90)	Non-CAA		Probable-CAA^c^ (*n* = 43)	*p*
				
		non-cSVD^a^ (*n* = 30)	cSVD^b^ (*n* = 17)		
**Demographics**					
Age (years), mean (SD)	73.5 (7.0)	69.7 (7.4)	77.7 (5.4)	74.4 (5.9)	b > a*** c > a**
Female, n (%)	45 (50)	16 (53.3)	8 (47.1)	21 (48.8)	0.898
Education (years), median [IQR]	16 [14–18]	16 [14–18]	16 [14–17]	17 [16–19]	0.064
**Vascular risk factors**					
Hypertension, n (%)^†^	55 (65.5)	18 (72.0)	11 (68.8)	26 (60.5)	0.599
Diabetes, n (%)^†^	11 (13.1)	2 (8.0)	5 (31.2)	4 (9.3)	0.071
Atrial fibrillation, n (%)^†^	4 (4.8)	1 (4.0)	1 (6.2)	2 (4.7)	1.000
Dyslipidemia, n (%)^†^	68 (81.0)	19 (76.0)	13 (81.2)	36 (83.7)	0.761
**Cognitive scores (z-scores)**					
MMSE, mean (SD)	−0.11 (1.43)	−0.18 (1.53)	0.51 (1.29)	−0.30 (1.38)	0.132
Memory, mean (SD)	−0.38 (1.25)	−0.14 (1.29)	−0.04 (1.19)	−0.67 (1.2)	0.092
Processing speed, mean (SD)	−0.07 (0.53)	−0.04 (0.50)	−0.08 (0.54)	−0.09 (0.55)	0.921
Language, mean (SD)	−0.34 (1.02)	−0.21 (0.81)	−0.27 (1.35)	−0.46 (1.02)	0.570
Executive function, median [IQR]	−0.26 [−0.8, 0.1]	−0.23 [−0.6, 0.2]	−0.07 [−0.6, 0.1]	−0.37 [−1.0, 0.1]	0.413
Gap MRIxNPT, months, median [IQR]	0 [−2.3, 0]	−1.05 [−3.4, 0]	−1.33 [−2.8, 0.2]	0 [−1.6, 0]	0.034^‡^
**Conventional MRI markers**					
Lobar CMB count, median [IQR]	0 [0, 13.5]	0 [0, 0]	0 [0, 0]	15 [3, 55]	c > a*** c > b***
cSS (presence), n (%)	14 (15.6)	0 (0)	0 (0)	14 (32.6)	c > a*** c > b***
Lacunes count, median [IQR]	0 [0, 1]	0 [0, 0]	1 [0, 1.5]	0 [0, 1]	b > a*** c > a***
Cortical CMI, count, median [IQR]	0 [0, 0]	0 [0, 0]	0 [0, 0]	0 [0, 1]	0.049^‡^
CSO PVS score, median [IQR]	2 [2, 3]	2 [1, 2]	2 [2, 2]	3 [2, 4]	c > a*** c > b**
BG PVS score, median [IQR]	2 [1, 2]	1 [1, 2]	2 [2, 3]	2 [1, 3]	b > a*** c > a***
nWMHV (% ICV), median [IQR]	0.2 [0.05, 0.8]	0.05 [0.01, 0.1]	0.66 [0.3, 1.2]	0.36 [0.14, 1.8]	b > a*** c > a***
nTBV (% ICV), mean (SD)	64.75 (4.26)	67.06 (4.46)	64.77 (3.26)	63.13 (3.76)	a > c***
**DTI global markers**					
PSMD (×10^–4^ mm^2^/s), median [IQR]	3.76 [3.32, 4.37]	3.30 [3.13, 3.60]	4.07 [3.51, 4.62]	4.06 [3.58, 4.79]	b > a*** c > a***
Average MD (×10^–4^ mm^2^/s), median [IQR]	8.44[7.86, 9.2]	7.98 [7.49, 8.61]	8.06 [7.75, 8.30]	9.19 [8.45, 9.83]	c > a*** c > b***
Average FA, mean (SD)	0.46 (0.04)	0.49 (0.04)	0.48 (0.03)	0.44 (0.04)	a > c*** b > c***
Total motion index, median [IQR]	−0.07 [−0.96, 1.26]	0.24[−1.07, 1.3]	0.21 [−0.66, 1.9]	−0.25 [−0.94, 0.6]	0.426
**DTI regional assessment**					
Frontal PSMD (×10^–4^ mm^2^/s), median [IQR]	3.14 [2.77, 3.50]	2.81 [2.52, 3.17]	3.26 [3.00, 3.89]	3.20 [3.03, 3.94]	b > a*** c > a***
Occipital PSMD (×10^–4^ mm^2^/s), median [IQR]	3.77 [3.23, 4.50]	3.23 [2.82, 3.70]	3.76 [3.53, 4.06]	4.44 [3.68, 5.00]	b > a* c > a***
Occipital-frontal PSMD gradient (×10^–4^ mm^2^/s), mean (SD)	0.76 (1.08)	0.43 (0.54)	0.45 (1.19)	1.11 (1.22)	c > a* c > b*
Frontal average MD (×10^–4^ mm^2^/s), median [IQR]	8.17 [7.74, 9.00]	7.84 [7.25, 8.38]	7.90 [7.60, 8.14]	8.98 [8.15, 9.55]	c > a*** c > b***
Occipital average MD (×10^–4^ mm^2^/s), median [IQR]	8.53 [8.14, 9.53]	8.27 [7.80, 8.51]	8.13 [7.78, 8.49]	9.35 [8.67, 9.81]	c > a*** c > b***
Occipital-frontal Average MD gradient (×10^–4^ mm^2^/s), mean (SD)	0.39 (0.45)	0.46 (0.27)	0.23 (0.40)	0.41 (0.55)	0.250
Frontal average FA, mean (SD)	0.42 (0.04)	0.45 (0.04)	0.43 (0.03)	0.40 (0.04)	a > c*** b > c***
Occipital average FA, mean (SD)	0.44 (0.05)	0.47 (0.04)	0.45 (0.03)	0.41 (0.04)	a > c*** b > c***
Occipital-frontal average FA gradient, median [IQR]	0.02 [0.01, 0.03]	0.03 [0.02, 0.03]	0.03 [0.00, 0.04]	0.02 [0.01, 0.03]	0.130

Kruskal Wallis and ANOVA were used to investigate differences across the three groups, as appropriate.

^†^Six missing values.

We reported the original *p*-values and, when significant differences were found, we ran FDR-adjusted pairwise comparisons: *FDR-adjusted *p* < 0.05.

**FDR-adjusted *p* < 0.01.

***FDR-adjusted *p* < 0.001.

^‡^No statistical significance in pairwise comparisons, after FDR-correction.

SD, standard deviation; IQR, interquartile range; MMSE, mini mental state examination; CMB, cerebral microbleeds; cSS, cortical superficial siderosis; CMI, cerebral microinfarcts; CSO-PVS, perivascular spaces in the centrum semiovale; BG-PVS, perivascular spaces in the basal ganglia; nWMHV, normalized white matter hyperintensity volume; nTBV, normalized total brain volume; CAA, cerebral amyloid angiopathy; cSVD, cerebral small vessel disease; PSMD, peak width of skeletonized mean diffusivity. Probable-CAA, patients fulfilling the modified Boston criteria for Probable CAA; cSVD, patients with neuroimaging markers of cSVD not attributable to CAA—presumed arteriolosclerosis; non-cSVD, patients without neuroimaging markers of cSVD.

^a^Non-cSVD group; ^b^cSVD group; ^C^Probable CAA group.

### Regional diffusion tensor imaging analyses

Controlling for age, occipital-frontal PSMD gradients were different across the three groups [*F*(2, 86) = 4.278, *p* = 0.017, partial η^2^ = 0.090]. Probable-CAA subjects presented higher occipital-frontal PSMD gradients in comparison to both cSVD (0.734 ± 0.30 × 10^–4^ mm^2^/s, *p* = 0.046) and non-cSVD (0.567 ± 0.26 × 10^–4^ mm^2^/s, *p* = 0.046) groups. There was a significant interaction between region (frontal x occipital) and group (non-cSVD, cSVD, probable-CAA) on PSMD values [*F*(2, 87) = 3.887, *p* = 0.024, partial η^2^ = 0.082, Figure 1B]. Frontal PSMD values, adjusted for age, were lower in the non-cSVD group in comparison to cSVD (mean difference ± standard error; FDR-adjusted *p*-values; −0.071 ± 0.03 × 10^–4^ mm^2^/s, *p* = 0.017) and probable-CAA (−0.070 ± 0.02 × 10^–4^ mm^2^/s, *p* = 0.004) groups, but were similar in the latter two (−0.001 ± 0.02 × 10^–4^ mm^2^/s, *p* = 0.955; Figure 1C). PSMD values from the occipital lobes, adjusted for age, were higher in probable-CAA in comparison to cSVD (0.069 ± 0.03 × 10^–4^ mm^2^/s, *p* = 0.026) and non-cSVD (0.117 ± 0.02 × 10^–4^ mm^2^/s, *p* < 0.001; Figure 1D) groups. Occipital PSMD values were higher than frontal values in probable-CAA [*F*(1, 42) = 46.059, *p* < 0.001, η^2^ = 0.523] and non-cSVD [*F*(1, 29) = 18.755, *p* < 0.001, partial η^2^ = 0.393] groups, but not in the cSVD group [*F*(1, 16) = 3.342, *p* = 0.086, partial η^2^ = 0.173; Figures 1F–H].

A similar analysis performed with MD data revealed: (1) controlling for age, occipital-frontal MD gradients did not differ across the three groups [*F*(2, 86) = 1.446, *p* = 0.241, partial η^2^ = 0.033]; (2) there was no significant interaction between region (frontal x occipital) and group (non-cSVD, cSVD, probable-CAA) on MD values [*F*(2, 87) = 1.782, *p* = 0.174, partial η^2^ = 0.039]; (3) the main effect of region showed a statistically significant difference in MD values at different lobes [*F*(1, 87) = 59.915, *p* < 0.001, partial η^2^ = 0.408], with higher values in the occipital lobe (0.019 ± 0.002 × 10^–4^ mm^2^/s, *p* < 0.001); (4) the main effect of group showed that there was a statistically significant difference in MD values between the three groups [*F*(2, 87) = 25.004, *p* < 0.001, partial η^2^ = 0.365], with higher values observed in the probable CAA groups compared to both cSVD (0.052 ± 0.01 × 10^–4^ mm^2^/s, *p* < 0.001), and non-cSVD samples (0.051 ± 0.01 × 10^–4^ mm^2^/s, *p* < 0.001).

With regards to FA data, we observed: (1) controlling for age, occipital-frontal FA gradients were similar across the three groups [*F*(2, 86) = 0.769, *p* = 0.467, partial η^2^ = 0.018]; (2) there was no significant interaction between region (frontal × occipital) and group (non-cSVD, cSVD, probable-CAA) on MD values [*F*(2, 87) = 0.574, *p* = 0.565, partial η^2^ = 0.013]; (3) the main effect of region showed a statistically significant difference in FA values at different lobes [*F*(1, 87) = 93.253, *p* < 0.001, partial η^2^ = 0.517], with higher values in the occipital lobe (0.02 ± 0.002, *p* < 0.001); (4) the main effect of group showed that there was a statistically significant difference in FA values between the three groups [*F*(2, 87) = 21.297, *p* < 0.001, partial η^2^ = 0.329], with lower values observed in the probable CAA groups compared to both cSVD (−0.041 ± 0.01, *p* < 0.001), and non-cSVD samples (−0.056 ± 0.01, *p* < 0.001).

### Relationship between diffusion tensor imaging markers and conventional MRI markers of cerebral small vessel diseases in cerebral amyloid angiopathy

**FIGURE 2:**
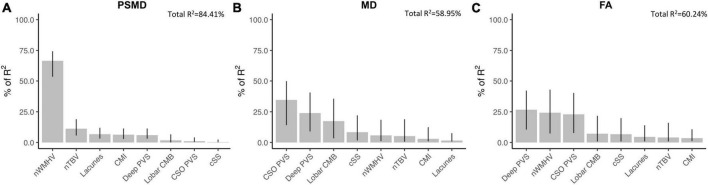
Associations between DTI metrics and conventional neuroimaging markers of cSVD in probable-CAA subjects. Bar graphs represent the relative contribution of each neuroimaging marker to explaining the variance in PSMD **(A)**, average skeletonized MD **(B)**, and average skeletonized FA **(C)** values among probable-CAA subjects. All neuroimaging markers were entered as independent variables in multiple linear regression models, with each DTI metric as the dependent variable. We applied a model decomposition method available in R package “Relaimpo” to compute the LMG metric. Metrics are normalized to sum to 100%. Lines represent 95% confidence intervals after 1,000 bootstrapping replications.

Among probable-CAA subjects (*n* = 43), regression models with PSMD as the dependent variable, adjusting for age and correcting for multiple comparisons, revealed that higher PSMD was associated with higher number of lacunes (Standardized beta coefficient [95% confidence interval]; FDR-adjusted *p*-value; β [95% CI] = 0.38[0.09, 0.67]; *p* = 0.022), nWMHV (β [95% CI] = 0.86[0.69, 1.03]; *p* < 0.001), and cortical CMI (β [95% CI] = 0.40[0.11, 0.69]; *p* = 0.022); and with lower nTBV (β [95% CI] = −0.48[−0.82, −0.14]; *p* = 0.022) (Supplementary Table 1). Among all MRI markers, nWMHV alone explained more than 65% of PSMD’s variance in the model (Figure 2A). Similar analyses with average skeletonized MD and FA values revealed associations with BG PVS, CSO PVS, lobar cerebral microbleeds, and nWMHV (Figures 2B,C and Supplementary Table 1).

### Relationship between diffusion tensor imaging markers and cognitive functions in cerebral amyloid angiopathy

**FIGURE 3:**
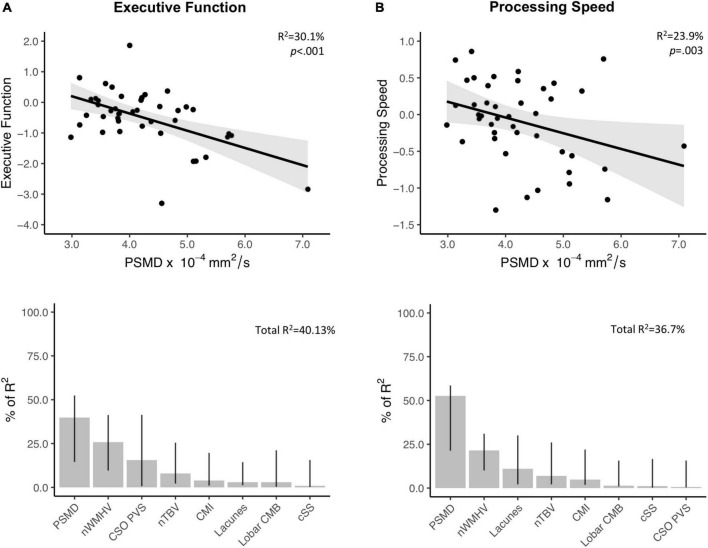
Associations between PSMD and performance in executive function and processing speed. Upper panel: Results from simple linear regression models between PSMD values and executive function **(A. Above)** and processing speed **(B. Above)** in probable-CAA subjects. 95% confidence intervals are depicted in gray. The provided R^2^ and *p*-values reflect the obtained independent predictive of PSMD with regards to cognitive scores adjusted for the interval between MRI and neuropsychological tests. Lower panel: Bar graphs representing the relative contribution of each neuroimaging marker to explaining the variance in executive **(A. Below)** and processing speed scores **(B. Below)**, using the LMG metric computed with the R package “Relaimpo”. Metrics are normalized to sum to 100%. Lines represent 95% confidence intervals after 1,000 bootstrapping replications.

In the probable-CAA group, linear regression models corrected for time interval between MRI and NPT revealed that higher PSMD values are associated with worse performance in executive function (Standardized beta; FDR-adjusted *p*-value; β [95% CI] = −0.58[−0.87, −0.30]; *p* = 0.002) and processing speed (β [95% CI] = −0.46[−0.76, −0.17]; *p* = 0.03) (Supplementary Table 2). PSMD was not associated with other cognitive domains (Supplementary Table 2). Among all neuroimaging features, other than PSMD, only nWMHV showed an association with cognition, after correction for multiple comparisons (executive function; β [95% CI] = −0.54[−0.84, −0.24; *p* = 0.004]). MD and FA were not associated with performance in any of the cognitive domains investigated (Supplementary Table 2). In multiple regression models applying a model decomposition method, PSMD contributed more than the other conventional MRI markers in explaining cognitive variance in the domains of executive function and processing speed (Figure 3).

As an exploratory analysis, suggested during the revision process, we investigated the cognitive associations of lobe-specific DTI metrics and their occipital-frontal gradients. After correcting for multiple comparisons, only global and frontal PSMD values were significantly associated with cognitive performance (i.e., executive function) (Supplementary Table 3).

## Discussion

In this study in a well-characterized memory-clinic cohort, we aimed to further investigate PSMD’s role as neuroimaging marker in the context of CAA. First, we observed that PSMD was higher in individuals with cSVD than in those without evidence of cSVD but similar between different cSVD phenotypes (probable CAA and arteriolosclerosis). Second, taking a regional approach, we observed higher occipital-frontal PSMD gradients in probable-CAA than non-cSVD and cSVD subjects and found a significant interaction between diagnosis and regional PSMD variation. Third, in probable-CAA, PSMD differed from classical DTI metrics in terms of neuroimaging and cognitive associations. nWMHV was the main predictor of PSMD values, whereas MD and FA were more strongly associated with other markers, particularly with perivascular spaces. PSMD was associated with executive function and processing speed in subjects with probable CAA and explained more variance than conventional neuroimaging markers. MD and FA were not associated with cognitive performance.

Higher PSMD values observed in probable-CAA and cSVD subjects support the idea that PSMD, like other DTI measures, is particularly sensitive to cSVD-related abnormalities (Baykara et al., 2016; Finsterwalder et al., 2020; Low et al., 2020). PSMD values in probable-CAA and cSVD subjects from our study are comparable to those previously reported in other cSVD samples: CADASIL (4.5–5.47 × 10^4^ mm^2^/s) (Baykara et al., 2016; Vinciguerra et al., 2018), memory-clinic subjects with sporadic cSVD (4.24 × 10^4^ mm^2^/s)(Baykara et al., 2016), and subjects with WM lesions and VCID (4.51 × 10^4^ mm^2^/s) (Wei et al., 2019). McCreary et al. (2020) reported slightly higher PSMD among 34 CAA subjects (4.97 × 10^4^ mm^2^/s), which could be explained by the presence of symptomatic ICH in that cohort. As expected, like other DTI-based techniques, global PSMD values are similar across different cSVD phenotypes and do not appear specific to any WM disease.

Though PSMD was originally developed as a global measure of WM integrity, our study supports that it can be successfully applied in a spatially oriented way, opening up interesting research possibilities. Expanding on existing research (Beaudet et al., 2020; Low et al., 2020), we calculated lobe-specific measures of PSMD, investigating between-group differences. We found that the degree to which PSMD values increased posteriorly was higher among probable-CAA compared to non-cSVD and cSVD subjects, indicating that there is a significant interaction between diagnosis and anteroposterior PSMD variations. The higher occipital-frontal PSMD gradients observed in the probable-CAA sample suggest that WM microstructural damage may be more severe in the posterior areas of the brain. This is consistent with several histopathological (Vinters and Gilbert, 1983; van Veluw et al., 2019a) and imaging (Smith et al., 2004; Rosand et al., 2005; Thanprasertsuk et al., 2014; Schouten et al., 2019) studies showing a posterior predilection of CAA pathology. This finding reflects a pattern of brain injury consistent with vascular amyloid deposition and could potentially provide insights into the predominant type of underlying microvascular pathology. Interestingly, we did not observe similar findings with MD or FA. That is, occipital-frontal MD and FA gradients did not differ between the groups. We also did not observe a significant interaction between region and group on these classical DTI metrics. Only PSMD displayed a spatial variation in line with the expected anteroposterior gradient of CAA pathology. This finding implies that different DTI metrics could show different sensitivity to underlying pathological changes, and could help explain their contrasting cognitive associations.

To further assess whether PSMD captures similar tissue abnormalities compared to average MD and FA, we investigated the relationship between conventional MRI markers of cSVD and each DTI metric. While PSMD is mainly associated with nWMHV, MD and FA strongly correlate with other markers, especially with PVS. Importantly, average MD and FA are central tendency measures, whereas PSMD is a dispersion metric (Beaudet et al., 2020). This difference could potentially explain the distinct neuroimaging associations observed. Furthermore, the degree to which these markers are influenced by specific technical parameters from the DTI protocol, such as the b-value, is uncertain and could potentially impact their ability to detect specific underlying tissue changes (Hui et al., 2010). The strong associations between PVS and classical DTI metrics align with recent observations that MD and FA changes in cSVD are mainly driven by increased water content in the extracellular compartment (named free-water), which includes the perivascular space (Duering et al., 2018). The exclusive and robust relationship between PSMD and non-hemorrhagic markers of cSVD, especially with WMH, has also been reported before (McCreary et al., 2020; Raposo et al., 2021). Ischemic markers are thought to cause structural disruption beyond the core lesions (van Veluw et al., 2017) and have been consistently associated with different DTI measures (Auriel et al., 2014; Reijmer et al., 2015a) and with cognitive function (van Dalen et al., 2015) in other cohorts.

Other DWI-based measures, like FA, apparent diffusion coefficient, and brain network measures, have been linked to loss of microstructural integrity in CAA (Salat et al., 2006; Viswanathan et al., 2008; Reijmer et al., 2015a), and some were also associated with cognitive performance (Viswanathan et al., 2008; Reijmer et al., 2015a; McCreary et al., 2020). Among these, only brain network analysis has been shown to reflect the anteroposterior gradient of CAA pathology, and, interestingly, it correlated with the same cognitive domains as PSMD (executive function and processing speed) (Reijmer et al., 2015a). These domains are commonly affected in VCID, which could imply that vascular pathology is likely the primary mechanism involved in the diffusion abnormalities captured by PSMD. Though it has been advocated that PSMD could be a specific or exclusive marker of processing speed (Smith and Beaudin, 2018), more recent studies with cSVD samples (Wei et al., 2019) and population-based cohorts (Deary et al., 2019; Low et al., 2020) found associations with other cognitive domains. It is noteworthy that PSMD outperformed both conventional and diffusion-based MRI markers of cSVD in our CAA cohort, which is consistent with findings from other groups (Baykara et al., 2016). Interestingly, in an exploratory analysis, while frontal PSMD values correlated with cognitive performance, occipital PSMD values did not. It is possible that frontal areas could be driving the associations observed between global PSMD and executive function/processing speed. This interpretation is in line with studies that support a central role of frontal regions, such as the dorsolateral prefrontal cortex, in orchestrating executive functions (Panikratova et al., 2020). Most conventional markers did not correlate with cognition in our sample, adding to their previously reported weak and inconsistent associations (Reijmer et al., 2015b). Though average MD and FA were not associated with cognition in our CAA sample, McCreary et al. (2020) found MD values to be associated with processing speed (*p* = 0.004) in a smaller sample of CAA patients. These conflicting results could potentially be due to the presence of symptomatic ICH in the Canadian cohort, which could significantly impact MD values and their cognitive associations.

Importantly, PSMD’s histopathological correlates have not yet been investigated. van Veluw et al. (2019b) found, in an *ex vivo* study of CAA patients, that MD values correlate with tissue rarefaction, myelin density, and WM microinfarcts. PSMD and mean MD values are both increased in CAA subjects and could reflect somewhat similar histopathological changes (McCreary et al., 2020). However, the distinct neuroimaging associations and spatial variation patterns observed between PSMD, and classical DTI metrics raise the possibility that they might be capturing slightly different histopathological abnormalities. Therefore, future studies investigating PSMD’s histological correlates are warranted and could improve the interpretation and biological understanding of this new and promising marker.

Our study has limitations. First, the cross-sectional design limits causal inference, calling for future longitudinal studies. Second, in this single-center study, we included only non-demented subjects from a memory-clinic setting, which accounts for a very specific CAA-profile and limits generalizability. Nonetheless, we chose to restrict recruitment from stroke-center subjects to avoid combining different phenotypes of CAA (Charidimou et al., 2015) in the same sample. By excluding demented patients, we aimed to capture earlier steps in the evolution of CAA and reduce the influence of concomitant AD pathology in our findings. The unavailability of cerebral spinal fluid (CSF) and positron emission tomography (PET) markers precluded a more detailed etiological characterization of the groups, based solely on MRI criteria.

## Conclusion

In conclusion, we provided evidence on PSMD’s promising role as a measure of both global and regional WM injury in CAA. Although global PSMD values are similarly increased in different forms of cSVD, regional PSMD analyses may capture disease-specific spatial variations. PSMD outperforms conventional and DTI-based markers as a cognitive biomarker in CAA and shows a distinct profile of neuroimaging correlates compared to MD and FA. Future research should investigate PSMD’s histopathological correlates and its performance in larger multi-center longitudinal CAA cohorts.

## Data availability statement

The raw data supporting the conclusions of this article will be made available by the authors, without undue reservation.

## Ethics statement

The studies involving human participants were reviewed and approved by the Institutional Review Board of the Massachusetts General Hospital (MGH). The patients/participants provided their written informed consent to participate in this study.

## Author contributions

MZ: study concept or design, major role in the acquisition of data, analysis or interpretation of data, and drafting/revision of the manuscript. DS: major role in the acquisition of data, analysis or interpretation of data, and drafting/revision of the manuscript. MH: major role in the acquisition of data and drafting/revision of the manuscript. NR, VP, MB, LS, QL, SV, ME, AC, MG, MD, AS, and OP-N: analysis or interpretation of data and drafting/revision of the manuscript. AV: study concept or design, analysis or interpretation of data, and drafting/revision of the manuscript. All authors contributed to the article and approved the submitted version.

## Footnotes

1
http://www.psmd-marker.com

2
https://surfer.nmr.mgh.harvard.edu

3
www.statistical-modelling.de/lst.html

## References

[B1] Andersson J. L. R. Sotiropoulos S. N. (2015). Non-parametric representation and prediction of single- and multi-shell diffusion-weighted MRI data using gaussian processes. *Neuroimage* 122 166–176. 10.1016/j.neuroimage.2015.07.067 26236030 PMC4627362

[B2] Arvanitakis Z. Leurgans S. E. Wang Z. Wilson R. S. Bennett D. A. Schneider J. A. (2011). Cerebral amyloid angiopathy pathology and cognitive domains in older persons. *Ann. Neurol.* 69 320–327. 10.1002/ana.22112 21387377 PMC3228518

[B3] Auriel E. Edlow B. L. Reijmer Y. D. Fotiadis P. Ramirez-Martinez S. Ni J. (2014). Microinfarct disruption of white matter structure. *Neurology* 83 182–188. 10.1212/wnl.0000000000000579 24920857 PMC4117171

[B4] Baykara E. Gesierich B. Adam R. Tuladhar A. M. Biesbroek J. M. Koek H. L. (2016). A novel imaging marker for small vessel disease based on skeletonization of white matter tracts and diffusion histograms. *Ann. Neurol.* 80 581–592. 10.1002/ana.24758 27518166

[B5] Beaudet G. Tsuchida A. Petit L. Tzourio C. Caspers S. Schreiber J. (2020). Age-related changes of peak width skeletonized mean diffusivity (PSMD) across the adult lifespan: A multi-cohort study. *Front. Psychiatry* 11:342. 10.3389/fpsyt.2020.00342 32425831 PMC7212692

[B6] Brandt J. (1991). The hopkins verbal learning test: Development of a new memory test with six equivalent forms. *Clin. Neuropsychol.* 5 125–142. 10.1080/13854049108403297

[B7] Charidimou A. Martinez-Ramirez S. Shoamanesh A. Oliveira-Filho J. Frosch M. Vashkevich A. (2015). Cerebral amyloid angiopathy with and without hemorrhage. *Neurology* 84 1206–1212. 10.1212/wnl.0000000000001398 25716356 PMC4366086

[B8] Corrigan J. D. Hinkeldey N. S. (1987). Relationships between parts a and b of the trail making test. *J. Clin. Psychol.* 43 402–409. 10.1002/1097-4679(198707)43:4<402::aid-jclp2270430411>3.0.co;2-e 3611374

[B9] Crum R. M. Anthony J. C. Bassett S. S. Folstein M. F. (1993). Population-based norms for the mini-mental state examination by age and educational level. *JAMA* 269 2386–2391. 10.1001/jama.1993.03500180078038 8479064

[B10] Dale A. M. Fischl B. Sereno M. I. (1999). Cortical surface-based analysis I. segmentation and surface reconstruction. *Neuroimage* 9 179–194. 10.1006/nimg.1998.0395 9931268

[B11] Deary I. J. Ritchie S. J. Maniega S. M. Cox S. R. Hernández M. C. V. Luciano M. (2019). Brain peak width of skeletonized mean diffusivity (PSMD) and cognitive function in later life. *Front. Psychiatry* 10:524. 10.3389/fpsyt.2019.00524 31402877 PMC6676305

[B12] Duering M. Finsterwalder S. Baykara E. Tuladhar A. M. Gesierich B. Konieczny M. J. (2018). Free water determines diffusion alterations and clinical status in cerebral small vessel disease. *Alzheimer’s Dement.* 14 764–774. 10.1016/j.jalz.2017.12.007 29406155 PMC5994358

[B13] Fastenau P. S. Denburg N. L. Mauer B. A. (1998). Parallel short forms for the Boston naming test: Psychometric properties and norms for older adults. *J. Clin. Exp. Neuropsychol.* 20 828–834. 10.1076/jcen.20.6.828.1105 10484693

[B14] Fazekas F. Chawluk J. Alavi A. Hurtig H. Zimmerman R. (1987). MR signal abnormalities at 1.5 T in Alzheimer’s dementia and normal aging. *Am. J. Roentgenol.* 149 351–356. 10.2214/ajr.149.2.351 3496763

[B15] Finsterwalder S. Vlegels N. Gesierich B. Caballero M. ÁA. Weaver N. A. Franzmeier N. (2020). Small vessel disease more than alzheimer’s disease determines diffusion MRI alterations in memory clinic patients. *Alzheimer’s Dement.* 16 1504–1514. 10.1002/alz.12150 32808747 PMC8102202

[B16] Grömping U. (2006). Relative importance for linear regression in R : The package relaimpo. *J. Stat. Softw.* 17 1–27. 10.18637/jss.v017.i01

[B17] Hui E. S. Cheung M. M. Chan K. C. Wu E. X. (2010). B-value dependence of DTI quantitation and sensitivity in detecting neural tissue changes. *Neuroimage* 49 2366–2374. 10.1016/j.neuroimage.2009.10.022 19837181

[B18] Jenkinson M. Beckmann C. F. Behrens T. E. J. Woolrich M. W. Smith S. M. (2012). FSL. *Neuroimage* 62 782–790. 10.1016/j.neuroimage.2011.09.015 21979382

[B19] Joy S. Kaplan E. Fein D. (2004). Speed and memory in the WAIS-III digit symbol—coding subtest across the adult lifespan. *Arch. Clin. Neuropsychol.* 19 759–767. 10.1016/j.acn.2003.09.009 15288329

[B20] Lam B. Y. K. Leung K. T. Yiu B. Zhao L. Biesbroek J. M. Au L. (2019). Peak width of skeletonized mean diffusivity and its association with age-related cognitive alterations and vascular risk factors. *Alzheimer’s Dement. Diagn. Assess. Dis. Monit.* 11 721–729. 10.1016/j.dadm.2019.09.003 31700990 PMC6829102

[B21] Lindeman R. H. Merenda P. F. Gold R. Z. (1980). *Introduction to Bivariate and Multivariate Analysis.* Northbrook: Foresman.

[B22] Linn J. Halpin A. Demaerel P. Ruhland J. Giese A. D. Dichgans M. (2010). Prevalence of superficial siderosis in patients with cerebral amyloid angiopathy. *Neurology* 74 1346–1350. 10.1212/wnl.0b013e3181dad605 20421578 PMC2875936

[B23] Low A. Mak E. Stefaniak J. D. Malpetti M. Nicastro N. Savulich G. (2020). Peak width of skeletonized mean diffusivity as a marker of diffuse cerebrovascular damage. *Front. Neurosci.* 14:238. 10.3389/fnins.2020.00238 32265640 PMC7096698

[B24] Mack W. J. Freed D. M. Williams B. W. Henderson V. W. (1992). Boston naming test: Shortened versions for use in alzheimer’s disease. *J. Gerontol.* 47 154–158. 10.1093/geronj/47.3.p154 1573197

[B25] McCreary C. R. Beaudin A. E. Subotic A. Zwiers A. M. Alvarez A. Charlton A. (2020). Cross-sectional and longitudinal differences in peak skeletonized white matter mean diffusivity in cerebral amyloid angiopathy. *Neuroimage Clin.* 27:102280. 10.1016/j.nicl.2020.102280 32521475 PMC7284130

[B26] Panikratova Y. R. Vlasova R. M. Akhutina T. V. Korneev A. A. Sinitsyn V. E. Pechenkova E. V. (2020). Functional connectivity of the dorsolateral prefrontal cortex contributes to different components of executive functions. *Int. J. Psychophysiol.* 151, 70–79. 10.1016/j.ijpsycho.2020.02.013 32109499

[B27] Pasi M. Charidimou A. Boulouis G. Auriel E. Ayres A. Schwab K. M. (2018). Mixed-location cerebral hemorrhage/microbleeds. *Neurology* 90:e119–e126. 10.1212/wnl.0000000000004797 29247070 PMC5772153

[B28] Potter G. M. Chappell F. M. Morris Z. Wardlaw J. M. (2015). Cerebral perivascular spaces visible on magnetic resonance imaging: Development of a qualitative rating scale and its observer reliability. *Cerebrovasc. Dis.* 39 224–231. 10.1159/000375153 25823458 PMC4386144

[B29] Raposo N. Zotin M. C. Z. Schoemaker D. Xiong L. Fotiadis P. Charidimou A. (2021). Peak width of skeletonized mean diffusivity as neuroimaging biomarker in cerebral amyloid angiopathy. *Am. J. Neuroradiol*. 42 875–881. 10.3174/ajnr.a7042 33664113 PMC8115367

[B30] Reijmer Y. D. Fotiadis P. Martinez-Ramirez S. Salat D. H. Schultz A. Shoamanesh A. (2015a). Structural network alterations and neurological dysfunction in cerebral amyloid angiopathy. *Brain* 138 179–188. 10.1093/brain/awu316 25367025 PMC4285191

[B31] Reijmer Y. D. van Veluw S. J. Greenberg S. M. (2015b). Ischemic brain injury in cerebral amyloid angiopathy. *J. Cereb. Blood Flow Metab.* 36 40–54. 10.1038/jcbfm.2015.88 25944592 PMC4758563

[B32] Rosand J. Muzikansky A. Kumar A. Wisco J. J. Smith E. E. Betensky R. A. (2005). Spatial clustering of hemorrhages in probable cerebral amyloid angiopathy. *Ann. Neurol.* 58 459–462. 10.1002/ana.20596 16130107

[B33] Salat D. H. Smith E. E. Tuch D. S. Benner T. Pappu V. Schwab K. M. (2006). White matter alterations in cerebral amyloid angiopathy measured by diffusion tensor imaging. *Stroke* 37 1759–1764. 10.1161/01.str.0000227328.86353.a7 16763176

[B34] Sánchez-Cubillo I. Periáñez J. A. Adrover-Roig D. Rodríguez-Sánchez J. M. Ríos-Lago M. Tirapu J. (2009). Construct validity of the trail making test: Role of task-switching, working memory, inhibition/interference control, and visuomotor abilities. *J. Int. Neuropsychol. Soc.* 15 438–450. 10.1017/s1355617709090626 19402930

[B35] Schmidt P. (2017). *Bayesian Inference For Structured Additive Regression Models For Large-Scale Problems With Applications To Medical Imaging*, Ph.D thesis, München: Universitätsbibliothek der Ludwig-Maximilians-Universität.

[B36] Schouten T. M. de Vos F. van Rooden S. Bouts M. J. R. J. van Opstal A. M. Feis R. A. (2019). Multiple approaches to diffusion magnetic resonance imaging in hereditary cerebral amyloid angiopathy mutation carriers. *J. Am. Heart Assoc.* 8:e011288. 10.1161/jaha.118.011288 30717612 PMC6405585

[B37] Smith E. E. Beaudin A. E. (2018). New insights into cerebral small vessel disease and vascular cognitive impairment from MRI. *Curr. Opin. Neurol.* 31 36–43. 10.1097/wco.0000000000000513 29084064

[B38] Smith E. E. Biessels G. J. Guio F. D. Leeuw F. E. Duchesne S. Düring M. (2019). Harmonizing brain magnetic resonance imaging methods for vascular contributions to neurodegeneration. *Alzheimer’s Dement. Diagn. Assess. Dis. Monit.* 11 191–204. 10.1016/j.dadm.2019.01.002 30859119 PMC6396326

[B39] Smith E. E. Gurol M. E. Eng J. A. Engel C. R. Nguyen T. N. Rosand J. (2004). White matter lesions, cognition, and recurrent hemorrhage in lobar intracerebral hemorrhage. *Neurology* 63 1606–1612. 10.1212/01.wnl.0000142966.22886.20 15534243

[B40] Smith S. M. (2002). Fast robust automated brain extraction. *Hum. Brain Mapp.* 17 143–155. 10.1002/hbm.10062 12391568 PMC6871816

[B41] Smith S. M. Jenkinson M. Johansen-Berg H. Rueckert D. Nichols T. E. Mackay C. E. (2006). Tract-based spatial statistics: Voxelwise analysis of multi-subject diffusion data. *Neuroimage* 31 1487–1505. 10.1016/j.neuroimage.2006.02.024 16624579

[B42] Thanprasertsuk S. Martinez-Ramirez S. Pontes-Neto O. M. Ni J. Ayres A. Reed A. (2014). Posterior white matter disease distribution as a predictor of amyloid angiopathy. *Neurology* 83 794–800. 10.1212/wnl.0000000000000732 25063759 PMC4155043

[B43] Tombaugh T. N. (2004). Trail making test A and B: Normative data stratified by age and education. *Arch. Clin. Neuropsychol.* 19 203–214. 10.1016/s0887-6177(03)00039-8 15010086

[B44] Tombaugh T. N. Kozak J. Rees L. (1999). Normative data stratified by age and education for two measures of verbal fluency fas and animal naming. *Arch. Clin. Neuropsychol.* 14 167–177. 10.1016/s0887-6177(97)00095-4 14590600

[B45] van Dalen J. W. Scuric E. E. M. Veluw S. J. van Caan M. W. A. Nederveen A. J. (2015). Cortical microinfarcts detected *in vivo* on 3 tesla MRI. *Stroke* 46 255–257. 10.1161/strokeaha.114.007568 25468879

[B46] van Veluw S. J. Scherlek A. A. Freeze W. M. Telgte A. Kouwe A. J. Bacskai B. J. (2019a). Different microvascular alterations underlie microbleeds and microinfarcts. *Ann. Neurol.* 86 279–292. 10.1002/ana.25512 31152566 PMC8722100

[B47] van Veluw S. J. Reijmer Y. D. van der Kouwe A. J. Charidimou A. Riley G. A. Leemans A. (2019b). Histopathology of diffusion imaging abnormalities in cerebral amyloid angiopathy. *Neurology* 92:e933–e943. 10.1212/wnl.0000000000007005 30700595 PMC6404469

[B48] van Veluw S. J. Shih A. Y. Smith E. E. Chen C. Schneider J. A. Wardlaw J. M. (2017). Detection, risk factors, and functional consequences of cerebral microinfarcts. *Lancet Neurol.* 16 730–740. 10.1016/s1474-4422(17)30196-5 28716371 PMC5861500

[B49] Vinciguerra C. Giorgio A. Zhang J. Donato I. D. Stromillo M. L. Brocci R. T. (2018). Peak width of skeletonized mean diffusivity (PSMD) as marker of widespread white matter tissue damage in multiple sclerosis. *Mult. Scler. Relat. Disord.* 27 294–297. 10.1016/j.msard.2018.11.011 30448470

[B50] Vinters H. V. Gilbert J. J. (1983). Cerebral amyloid angiopathy: Incidence and complications in the aging brain. II. The distribution of amyloid vascular changes. *Stroke* 14 924–928. 10.1161/01.str.14.6.924 6658996

[B51] Viswanathan A. Greenberg S. M. (2011). Cerebral amyloid angiopathy in the elderly. *Ann. Neurol.* 70 871–880. 10.1002/ana.22516 22190361 PMC4004372

[B52] Viswanathan A. Patel P. Rahman R. Nandigam R. N. K. Kinnecom C. Bracoud L. (2008). Tissue microstructural changes are independently associated with cognitive impairment in cerebral amyloid angiopathy. *Stroke* 39 1988–1992. 10.1161/strokeaha.107.509091 18436874 PMC2698787

[B53] Wardlaw J. M. Smith E. E. Biessels G. J. Cordonnier C. Fazekas F. Frayne R. (2013). Neuroimaging standards for research into small vessel disease and its contribution to ageing and neurodegeneration. *Lancet Neurol.* 12 822–838. 10.1016/s1474-4422(13)70124-8 23867200 PMC3714437

[B54] Wechsler D. (1987). *Wechsler Memory Scale-Revised Manual.* San Antonio: The Psychological Corporation.

[B55] Wei N. Deng Y. Yao L. Jia W. Wang J. Shi Q. (2019). A neuroimaging marker based on diffusion tensor imaging and cognitive impairment due to cerebral white matter lesions. *Front. Neurol.* 10:81. 10.3389/fneur.2019.00081 30814973 PMC6381840

[B56] Weintraub S. Salmon D. Mercaldo N. Ferris S. Graff-Radford N. R. Chui H. (2009). The alzheimer’s disease centers’ Uniform Data Set (UDS). *Alzheimer Dis. Assoc. Disord.* 23 91–101. 10.1097/wad.0b013e318191c7dd 19474567 PMC2743984

[B57] Xiong L. Boulouis G. Charidimou A. Roongpiboonsopit D. Jessel M. J. Pasi M. (2017). Dementia incidence and predictors in cerebral amyloid angiopathy patients without intracerebral hemorrhage. *J. Cereb. Blood Flow Metab.* 38 241–249. 10.1177/0271678x17700435 28318355 PMC5951014

[B58] Yendiki A. Koldewyn K. Kakunoori S. Kanwisher N. Fischl B. (2014). Spurious group differences due to head motion in a diffusion MRI study. *Neuroimage* 88 79–90. 10.1016/j.neuroimage.2013.11.027 24269273 PMC4029882

