# Supplementary material for: Peak width of skeletonized mean diffusivity in cerebral amyloid angiopathy: Spatial signature, cognitive, and neuroimaging associations
Source: Front Neurosci. 2022 Nov 11;16:1051038. doi: 10.3389/fnins.2022.1051038 (PMC9693722; doi:10.3389/fnins.2022.1051038)
Supplement: Supplementary file 1 [file Table_1.docx]

**Supplementary Table 1. Associations between DTI metrics and conventional neuroimaging markers of cSVD in probable-CAA subjects.**

**Legend.** Linear regression models with global PSMD, MD, or FA as the dependent variable, adjusted for age. The provided standardized beta coefficients, confidence intervals, and original *p*-values reflect the obtained independent predictive of the listed MRI marker with regards to PSMD, MD or FA. *Statistically significant in models not corrected for multiple comparisons. †Statistically significant after FDR correction within each model (that is, by column of this table). Abbreviations: BG-PVS = perivascular spaces in the basal ganglia; CI = confidence interval; CMB = cerebral microbleeds; CMI = cortical cerebral microinfarcts; CSO-PVS = perivascular spaces in the centrum semiovale; cSS = cortical superficial siderosis; ICV = intracranial volume; nTBV = normalized total brain volume; nWMHV = normalized white matter hyperintensities volume; Std.Beta = standardized beta coefficient.

| **Probable CAA**  **n=43** | **PSMD** | | | | | **MD** | | | | | **FA** | | | | |
| --- | --- | --- | --- | --- | --- | --- | --- | --- | --- | --- | --- | --- | --- | --- | --- |
|  | **Std.Beta** | **95% CI** | | **R^2^** | ***p*** | **Std.Beta** | **95% CI** | | **R^2^** | ***p*** | **Std.Beta** | **95% CI** | | **R^2^** | ***p*** |
| **cSVD markers** |  | | | | |  | | | | |  | | | | |
| Lobar CMB, count | 0.159 | -0.153 | 0.472 | 0.060 | .310 | 0.403 | 0.111 | 0.694 | 0.182 | .008*† | -0.285 | -0.594 | -0024 | 0.082 | .069 |
| cSS, presence | 0.112 | -0.208 | 0.432 | 0.047 | .483 | 0.329 | 0.023 | 0.636 | 0.126 | .036* | -0.310 | -0.622 | 0.001 | 0.094 | .051 |
| Lacune, count | 0.381 | 0.091 | 0.671 | 0.180 | .011*† | 0.144 | -0.169 | 0.456 | 0.043 | .359 | -0.241 | -0.551 | 0.069 | 0.060 | .124 |
| nWMHV (%ICV) | 0.859 | 0.688 | 1.030 | 0.731 | <.001* | 0.352 | 0.046 | 0.657 | 0.139 | .025* | -0.551 | -0.829 | -0.274 | 0.289 | <.001*† |
| Cortical CMI, count | 0.398 | 0.109 | 0.687 | 0.192 | .008*† | 0.218 | -0.092 | 0.527 | 0.069 | .163 | -0.265 | -0.575 | 0.044 | 0.072 | .091 |
| CSO PVS, score | 0.214 | -0.101 | 0.529 | 0.078 | .178 | 0.584 | 0.319 | 0.850 | 0.346 | <.001* | -0.531 | -0.812 | -0.251 | 0.270 | <.001*† |
| BG PVS, score | 0.308 | -0.003 | 0.619 | 0.123 | .052 | 0.619 | 0.357 | 0.881 | 0.377 | <.001* | -0.619 | -0.885 | -0.352 | 0.356 | <.001*† |
| nTBV (%ICV) | -0.477 | -0.815 | -0.139 | 0.198 | .007*† | -0.345 | -0.702 | 0.012 | 0.108 | .058 | 0.324 | -0.039 | 0.687 | 0.077 | .079 |
