# Supplementary material for: Peak width of skeletonized mean diffusivity in cerebral amyloid angiopathy: Spatial signature, cognitive, and neuroimaging associations
Source: Front Neurosci. 2022 Nov 11;16:1051038. doi: 10.3389/fnins.2022.1051038 (PMC9693722; doi:10.3389/fnins.2022.1051038)
Supplement: Supplementary file 2 [file Table_2.DOCX]

**Supplementary Table 2. Associations between neuroimaging markers and performance in the domains of processing speed, executive function, language and memory in probable-CAA.**

**Legend.** Simple linear regression models with each cognitive score as the dependent variable and each neuroimaging marker as the independent marker, adjusted for the interval between MRI and NPT. The provided standardized beta coefficients and *p*-values reflect the obtained independent predictive of the listed MRI marker with regards to cognitive scores. Abbreviations: CI = confidence interval; CMB = cerebral microbleeds; CMI = cerebral microinfarcts; CSO-PVS = perivascular spaces in centrum semiovale; cSS = cortical superficial siderosis; nTBV = normalized total brain volume, nWMHV = normalized white matter hyperintensities volume; Std.Beta = standardized beta coefficient. * statistically significant in models not corrected for multiple comparisons. † statistically significant after FDR correction within each cognitive domain (that is, by column of this table).

| **Probable CAA**  **n=43** | **Processing Speed** | | | | | **Executive Function** | | | | | **Language** | | | | | **Memory function** | | | | |
| --- | --- | --- | --- | --- | --- | --- | --- | --- | --- | --- | --- | --- | --- | --- | --- | --- | --- | --- | --- | --- |
|  | **Std.β** | **95% CI** | | **R^2^** | ***p*** | **Std.β** | **95% CI** | | **R^2^** | ***p*** | **Std.β** | **95% CI** | | **R^2^** | ***p*** | **Std.β** | **95% CI** | | **R^2^** | ***p*** |
| Global PSMD (x 10^-4^ mm^2^/s) | -0.463 | -0.759 | -0.167 | 0.239 | .003*† | -0.581 | -0.865 | -0.297 | 0.301 | <.001*† | -0.291 | -0.613 | .031 | 0.098 | .075 | -0.025 | -0.362 | 0.311 | 0.016 | .879 |
| Global MD (x 10^-4^ mm^2^/s) | -0.278 | -0.616 | 0.060 | 0.110 | .104 | -0.040 | -0.398 | 0.318 | 0.003 | .824 | 0.048 | -0.306 | 0.402 | 0.024 | .786 | 0.065 | -0.290 | 0.420 | 0.018 | .714 |
| Global FA | 0.247 | -0.078 | 0.572 | 0.102 | .132 | 0.133 | -0.207 | 0.473 | 0.017 | .433 | 0.052 | -0.287 | 0.390 | 0.025 | .759 | -0.117 | -0.456 | 0.221 | 0.027 | .487 |
| Lobar CMB, count | -0.093 | -0.407 | 0.221 | 0.057 | .553 | 0.104 | -0.218 | 0.425 | 0.012 | .519 | -0.126 | -0.443 | 0.191 | 0.038 | .427 | 0.033 | -0.288 | 0.354 | 0.016 | .836 |
| cSS, presence | 0.015 | -0.297 | 0.327 | 0.049 | .925 | 0.074 | -0.245 | 0.393 | 0.007 | .642 | 0.065 | -0.251 | 0.380 | 0.027 | .681 | 0.010 | -0.308 | 0.327 | 0.015 | .952 |
| Lacune, count | 0.039 | -0.273 | 0.351 | 0.050 | .802 | -0.130 | -0.447 | 0.187 | 0.018 | .413 | 0.026 | -0.290 | 0.342 | 0.023 | .867 | 0.044 | -0.273 | 0.361 | 0.017 | .781 |
| nWMHV (%ICV) | -0.271 | -0.597 | 0.054 | 0.112 | .100 | -0.538 | -0.838 | -0.239 | 0.249 | .001*† | -0.374 | -0.694 | -0.054 | 0.142 | .023* | 0.039 | -0.303 | 0.382 | 0.016 | .818 |
| Cortical CMI, count | -0.103 | -0.415 | 0.208 | 0.059 | .505 | -0.152 | -0.468 | 0.165 | 0.024 | .340 | -0.168 | -0.480 | 0.145 | 0.051 | .284 | 0.022 | -0.296 | 0.340 | 0.016 | .890 |
| CSO PVS, score | -0.140 | -0.461 | 0.181 | 0.067 | .383 | 0.242 | -0.081 | 0.565 | 0.056 | .138 | -0.069 | -0.397 | 0.259 | 0.027 | .673 | 0.080 | -0.249 | 0.408 | 0.021 | .628 |
| nTBV (%ICV) | -0.007 | -0.321 | 0.308 | 0.049 | .965 | 0.298 | -0.009 | 0.606 | 0.089 | .057 | -0.033 | -0.352 | 0.285 | 0.024 | .834 | 0.040 | -0.280 | 0.360 | 0.017 | .802 |
