# Supplementary material for: Peak width of skeletonized mean diffusivity in cerebral amyloid angiopathy: Spatial signature, cognitive, and neuroimaging associations
Source: Front Neurosci. 2022 Nov 11;16:1051038. doi: 10.3389/fnins.2022.1051038 (PMC9693722; doi:10.3389/fnins.2022.1051038)
Supplement: Supplementary file 3 [file Table_3.DOCX]

**Supplementary Table 3. Associations between regional DTI metrics and performance in the domains of executive function, processing speed, language and memory in probable-CAA.**

**Legend.** Simple linear regression models with each cognitive score as the dependent variable and each DTI metric as the independent marker, adjusted for the interval between MRI and NPT. The provided standardized beta coefficients and *p*-values reflect the obtained independent predictive of the listed MRI marker with regards to cognitive scores. Abbreviations: CI = confidence interval; FA= fractional anisotropy; MD = mean diffusivity; PSMD = peak width of skeletonized mean diffusivity; Std.β = standardized beta coefficient. * statistically significant in models not corrected for multiple comparisons. † statistically significant after FDR correction within each cognitive domain (that is, by column of this table).

| **Probable CAA**  **n=43** | **Processing Speed** | | | | | **Executive Function** | | | | | **Language** | | | | | **Memory function** | | | | |
| --- | --- | --- | --- | --- | --- | --- | --- | --- | --- | --- | --- | --- | --- | --- | --- | --- | --- | --- | --- | --- |
|  | **Std.β** | **95% CI** | | **R^2^** | ***p*** | **Std.β** | **95% CI** | | **R^2^** | ***p*** | **Std.β** | **95% CI** | | **R^2^** | ***p*** | **Std.β** | **95% CI** | | **R^2^** | ***p*** |
| Global PSMD (x 10^-4^ mm^2^/s) | -0.463 | -0.759 | -0.167 | 0.239 | .003*† | -0.581 | -0.865 | -0.297 | 0.301 | <.001*† | -0.291 | -0.613 | 0.031 | 0.098 | .075 | -0.025 | -0.362 | 0.311 | 0.016 | .879 |
| Frontal PSMD (x 10^-4^ mm^2^/s) | -0.351 | -0.652 | -0.051 | 0.165 | .023* | -0.529 | -0.811 | 0.247 | 0.265 | <.001*† | -0.275 | -0.588 | 0.038 | 0.094 | .084 | -0.098 | -0.423 | 0.227 | 0.024 | .546 |
| Occipital PSMD (x 10^-4^ mm^2^/s) | -0.293 | -0.609 | 0.023 | 0.126 | .068 | -0.155 | -0.489 | 0.179 | 0.023 | .353 | -0.060 | -0.394 | 0.273 | 0.026 | .717 | 0.299 | -0.023 | 0.620 | 0.095 | .068 |
| Occipital-frontal PSMD gradient | -0.026 | -0.342 | 0.290 | 0.049 | .869 | 0.240 | -0.075 | 0.554 | 0.057 | .131 | 0.144 | -0.172 | 0.461 | 0.043 | .363 | 0.363 | 0.063 | 0.663 | 0.143 | .019* |
| Global MD (x 10^-4^ mm^2^/s) | -0.278 | -0.616 | 0.060 | 0.110 | .104 | -0.040 | -0.398 | 0.318 | 0.003 | .824 | 0.048 | -0.306 | 0.402 | 0.024 | .786 | 0.065 | -0.290 | 0.420 | 0.018 | .714 |
| Frontal MD ( x 10^-4^ mm^2^/s) | -0.241 | -0.566 | 0.085 | 0.099 | .143 | 0.010 | -0.333 | 0.352 | 0.001 | .955 | 0.023 | -0.316 | 0.362 | 0.023 | .890 | 0.056 | -0.284 | 0.396 | 0.018 | .740 |
| Occipital MD (x 10^-4^ mm^2^/s) | -0.162 | -0.496 | 0.173 | 0.071 | .334 | 0.060 | -0.287 | 0.406 | 0.004 | 0.730 | 0.117 | -0.224 | 0.458 | 0.034 | .492 | 0.080 | -0.263 | 0.423 | 0.021 | .641 |
| Occipital-frontal MD gradient | 0.104 | -0.207 | 0.414 | 0.059 | .504 | 0.064 | -0.225 | 0.383 | 0.005 | .686 | 0.121 | -0.193 | 0.434 | 0.037 | .442 | 0.030 | -0.287 | 0.348 | 0.016 | .848 |
| Global FA | 0.247 | -0.078 | 0.572 | 0.102 | .132 | 0.133 | -0.207 | 0.473 | 0.017 | .433 | 0.052 | -0.287 | 0.390 | 0.025 | .759 | -0.117 | -0.456 | 0.221 | 0.027 | .487 |
| Frontal FA | 0.266 | -0.051 | 0.582 | 0.113 | .097 | 0.109 | -0.225 | 0.443 | 0.012 | .512 | 0.082 | -0.249 | 0.413 | 0.029 | .619 | -0.152 | -0.481 | 0.178 | 0.036 | .358 |
| Occipital FA | 0.176 | -0.145 | 0.497 | 0.077 | .274 | 0.149 | -0.182 | 0.479 | 0.022 | .369 | 0.017 | -0.313 | 0.347 | 0.023 | .917 | -0.066 | -0.397 | 0.265 | 0.019 | .690 |
| Occipital-frontal FA gradient | -0.131 | -0.440 | 0.178 | 0.066 | .395 | 0.097 | -0.221 | 0.415 | 0.011 | .539 | -0.115 | -0.429 | 0.199 | 0.036 | .464 | 0.144 | -0.169 | 0.458 | 0.036 | .358 |
